# 2485: ETV6 represses Pax5 in early B-cell development

**DOI:** 10.1017/cts.2017.234

**Published:** 2018-05-10

**Authors:** Greg Kirkpatrick, Courtney Jones, Susan Fosmire, Christopher Porter, Jorge DiPaola

**Affiliations:** University of Colorado at Denver, Denver, CO, USA

## Abstract

OBJECTIVES/SPECIFIC AIMS: The goal of this project is to determine the role of ETV6 in early B-cell development and define how germline ETV6 mutations result in predisposition to leukemia. METHODS/STUDY POPULATION: Gene expression commons were queried for expression levels of Etv6 and Pax5 at different stages of hematopoiesis. Mouse bone marrow was isolated and fractioned into cells committed to the B cell lineage via B220+ and CD43+ staining by flow cytometry and then separated into the following fractions: Fraction A (CD24low, CD19−), Fraction B (CD19+, CD24+, BP1−), and Fraction C (CD19+ CD24+ BP1+). Wild-type or germline mutant P214L ETV6 were cloned in an MiG vector and expressed in Ba/F3 cells. ChIP-PCR was performed by cross-linking proteins to DNA with 1% formaldehyde for 10 minute at room temperature, followed by cell lysis with RIPA buffer. Lysates were sonicated to shear DNA to a length of 200–1000 base pairs, then Protein A agarose beads were used to clean and immunoprecipitate chromatin. RESULTS/ANTICIPATED RESULTS: We observed that Etv6 is highly expressed in hematopoietic stem and lymphoid progenitor cells through the pre-pro-B stage (FrA), but its expression is significantly reduced in fraction B and thereafter (*p*<0.0001). Etv6 expression decreases as B cells develop and is negatively correlated with Pax5 expression (*r* ^2^=0.9993; *p*=0.0167). We next confirmed the expression patterns of ETV6 and PAX5 during B cell development in human samples. We found that ETV6 expression was higher in the early B cell fraction (CD10+, CD34+, CD19−, and CD20−) compared to the pre-B cell fraction (CD10+, CD34−, CD19+, CD20−). Conversely, we observed that PAX5 expression was higher in the preB cell fraction compared with the early B cell fraction. In Ba/F3 cells expressing ETV6 constructs, ETV6, but not ETV6 P214L overexpression significantly decreased Pax5 expression (*p*≤0.05). ETV6 is associated with the proximal GGAA site 72 base pairs upstream of the Pax5 TSS, but not GGAA sites further from the TSS. In addition, the transcriptional repressors SIN3A and HDAC3 were detected on the same regions of the Pax5 locus. We detected association of ETV6, SIN3A, and HDAC3 with the proximal GGAA site upon expression of WT ETV6, but not ETV6 P214L. DISCUSSION/SIGNIFICANCE OF IMPACT: Our results provide a mechanism of interaction for ETV6 and PAX5, 2 genes often disrupted in B-cell leukemia. These findings are significant because PAX5 misregulation results in a B cell development halt, lineage infidelity, and leukemogenesis. In continuing our studies, we have generated a transgenic mouse endogenously expressing the ETV6 P214L mutation by CRISPR/Cas9 editing, and these mice appear to have a thrombocytopenic phenotype similar to that observed in patients carrying the ETV6 P214L mutation. These animals will be the focus of our continued investigation of the mechanism by which ETV6 germline mutation results in a predisposition to leukemia. Our ultimate goal is a comprehensive understanding of how this process may be targeted more efficiently in patients with both heritable and sporadic forms of leukemia involving ETV6.

